# Distally Based Lymphatic Microsurgical Preventive Healing Approach—A Modification of the Classic Approach

**DOI:** 10.1055/a-2336-0150

**Published:** 2024-08-06

**Authors:** Allen Wei-Jiat Wong, Nadia Hui Shan Sim, Coeway Boulder Thing, Wenxuan Xu, Hui Wen Chua, Sabrina Ngaserin, Shermaine Loh, Yee Onn Kok, Jia Jun Feng, Tan Woon Woon Pearlie, Benita Kiat-Tee Tan

**Affiliations:** 1 Plastic, Reconstructive & Aesthetic Surgery Service, Sengkang General Hospital, Singapore; 2 Department of Plastic Reconstructive and Aesthetic Surgery, Singapore General Hospital, Singapore; 3 Toulouse School of Economics, Université Toulouse 1 Capitole, Toulouse, France; 4 Department of General Surgery, Breast Service, Sengkang General Hospital, Singapore; 5 Lee Kong Chian School of Medicine, Nanyang Technological University, Singapore; 6 Department of Plastic and Reconstructive Surgery, Chang Gung Memorial Hospital, College of Medicine, Chang Gung University, Taipei, Taiwan

**Keywords:** lymphedema, breast cancer, microsurgery, lymphovenous anastomosis

## Abstract

The treatment of breast cancer has seen great success in the recent decade. With longer survivorship, more attention is paid to function and aesthetics as integral treatment components. However, breast cancer-related lymphedema (BCRL) remains a significant complication. Immediate lymphatic reconstruction is an emerging technique to reduce the risk of BCRL, the Lymphatic Microsurgical Preventive Healing Approach (LYMPHA) being the most widely used approach. Despite promising results, it is often difficult to find suitably sized recipient venules and perform the microanastomoses between mismatched vessels deep in the axilla. Moreover, high axillary venous pressure gradients and potential damage from radiotherapy may affect the long-term patency of the anastomoses. From an ergonomic point of view, performing lymphaticovenular anastomosis in the deep axilla may be challenging for the microsurgeon. In response to these limitations, we modified the technique by moving the lymphatic reconstruction distally—terming it distally based LYMPHA (dLYMPHA). A total of 113 patients underwent mastectomy with axillary clearance in our institution from 2018 to 2021. Of these, 26 underwent subsequent dLYMPHA (Group 2), whereas 87 did not (Group 1). In total, 17.2% (15 patients) and 3.84% (1 patient) developed BCRL in Groups 1 and 2, respectively ( *p* = 0.018). Lymphatics and recipient venules suitable for anastomoses can be reliably found in the distal upper limb with better size match. A distal modification achieves a more favorable lymphaticovenular pressure gradient, vessel match, and ergonomics while ensuring a comparably low BCRL rate.

## Introduction

Breast cancer-related lymphedema (BCRL) is a chronic, progressive disease that poses a substantial psychological and economic burden. 1 According to a recent systematic review, 14.1% of patients who undergo axillary lymph node dissection (ALND) develop BCRL. Those who undergo ALND and adjuvant radiation have a 33.4% incidence of BCRL. 2 With great successes in the treatment of lower extremity lymphedema, reconstructive surgeons are now turning their attention to refining reconstructive techniques and improving quality of life in the treatment of BCRL. The use of prophylactic lymphaticovenular anastomosis (LVA) after axillary clearance has been implemented to reduce the risk of BCRL. Lymphatic Microsurgical Preventive Healing Approach (LYMPHA) is the most widely used approach. 3 Despite promising results and concerns, it is often difficult to find suitably sized recipient venules and perform the microanastomoses between mismatched vessels deep in the axilla. Also, high axillary venous pressure gradients and potential damage from radiotherapy may affect the long-term patency of the anastomoses. 4 To mitigate against these risks, we modified the LYMPHA technique by shifting the immediate lymphatic reconstruction (ILR) distally in the upper limb, terming it the distally based LYMPHA technique (dLYMPHA).

## Idea

### Methods

With Centralized Institutional Review Board (CIRB) approval (CIRB Reference 2022/2619), we conducted a retrospective study on patients who had undergone ALND related to breast cancer in our institution from January 2018 to December 2021. Patients included in this study were women with breast cancer presenting with axillary metastasis requiring ALND. Informed consent was obtained from patients prior to publication of their clinical photos. As part of our institutional practice, dLYMPHA was offered to all patients requiring ALND during preoperative counselling. The inclusion criteria for dLYMPHA were patients who require ALND related to breast cancer. The exclusion criteria were patients who did not require ALND, presence of systemic metastasis, or skin malignancy of the upper limbs. Patients with less than 6 months of follow-up were also excluded from this study, to exclude temporary swelling from postoperative changes. A total of 113 patients underwent ALND at our institution. Of these, 87 patients underwent ALND only (Group 1) and 26 underwent ALND with dLYMPHA (Group 2; Table 1 ).

**Table 1:** Demographics and background of patients in the study, with ALND only (Group 1) versus ALND with dLYMPHA (Group 2)

	Group 1 ( *N* = 87)	Group 2 ( *N* = 26)	*p* -Value
Demographics			
Mean age (years)	59 (29–97) (SD: 13.0)	54 (35–79) (SD: 11.2)	0.0784
BMI	24.0 (15.1–43.3) (SD: 4.82)	25.2 (18.7–33.8) (SD: 4.12)	0.193
**Surgical characteristics**			
Skin-sparing mastectomy (%)	60 (69.0)	19 (73.1)	0.949
Nipple-sparing mastectomy (%)	10 (11.5)	4 (15.4)	0.953
Lumpectomy (%)	15 (17.2)	1 (3.84)	0.547
Chest wall resection (%)	2 (2.30)	2 (7.69)	0.400
Lymph nodes retrieved	14.2 (1–47)	18.2 (1–38)	0.002 a
Metastatic lymph nodes	4.01 (0–36)	2.65 (0–20)	0.161
**Oncologic treatment characteristics**			
Neoadjuvant chemotherapy (%)	37 (42.5%)	12 (46.2%)	0.746
Neoadjuvant radiotherapy (%)	0	0	NA
Adjuvant chemotherapy (%)	44 (50.6%)	22 (84.6%)	0.0005 a
Adjuvant radiotherapy (%)	57 (65.5%)	19 (73.1%)	0.476

Abbreviations: ALND, axillary lymph node dissection; BMI, body mass index; dLYMPHA, distally based Lymphatic Microsurgical Preventive Healing Approach; SD, standard deviation.

a Denotes a statistically significant result.

Limb circumference measurements are recorded by the same physiotherapist before surgery, at 3-month intervals postoperatively for the first year, and at 6-month intervals thereafter. Each limb was measured at 4 cm intervals and extrapolated to obtain an estimated volume of the limb. Lymphedema diagnosis is established when there is a 10% increase in volume and on lymphoscintigraphy more than 6 months after surgery.

Descriptive statistics were reported as the number (percentage or mean [standard deviation]). Statistical analysis and logistical regression analysis were performed with STATA Ver. 17 (StataCorp, College Station, TX). Significance was set at 0.05.

### Distally based Lymphatic Microsurgical Preventive Healing Approach Technique

**Fig. 1:**
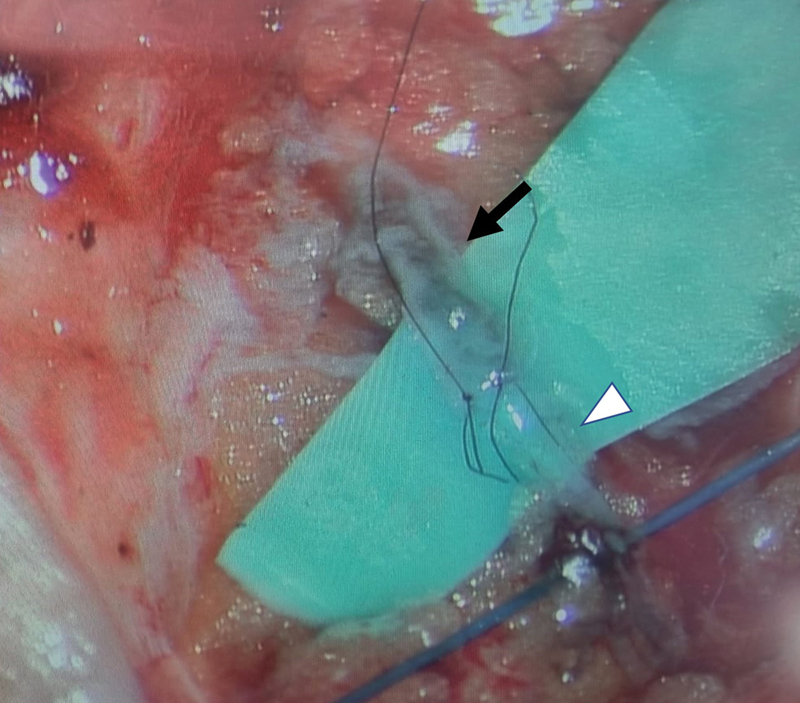
End-to-end LVA showing good flow of patent blue flowing across anastomosis. (black arrow: vein, white arrowhead: lymphatic vessel) LVA, lymphaticovenular anastomosis.

**Fig. 2:**
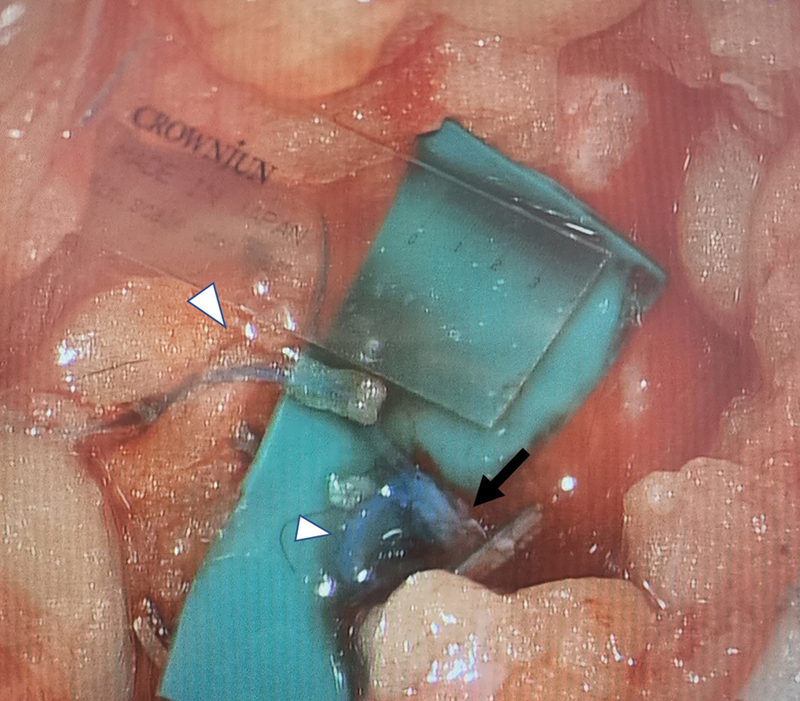
An LVA formed by anastomosing two lymphatic channels (white arrowhead) to one venule (black arrow), showing good flow of patent blue across anastomosis. LVA, lymphaticovenular anastomosis.

**Fig. 3:**
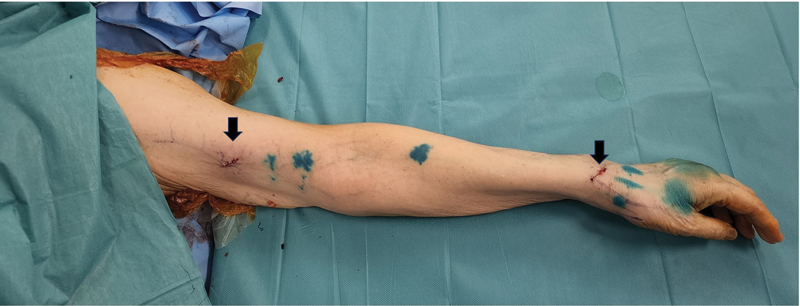
Left arm showing incisions (black arrows) near medial upper arm and over radial wrist.

**Fig. 4:**
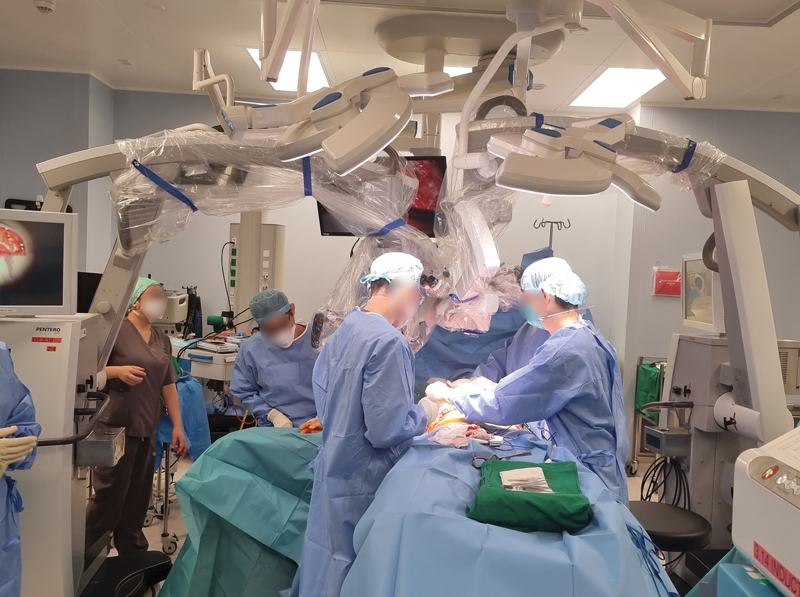
Intraoperative positioning for microscopes to allow simultaneous dLYMPHA and breast flap inset. dLYMPHA, distally based Lymphatic Microsurgical Preventive Healing Approach.

Indocyanine green (ICG) lymphangiography is performed on a table with a Stryker SPY-PHI device (Stryker Corporation, MI) to map lymphatic channels. Recipient venules are identified using an infrared vein finder (AccuVein Inc, NY). To improve the intraoperative visualization of lymphatic channels, we routinely inject patent blue into the first three dorsal webspace of the hand. Incisions are planned based on the location of the maximal confluence of the ICG and vein markings ( Figs. 1 2 3 ). The larger lymphatic vessels in the upper limb run close to the cephalic and basilic veins. 5 To ensure adequate decompression of both lymphatic systems, we will place a wrist incision on the radial wrist to capture the lymphatic vessels along the cephalic vein, and a bicipital grove incision to capture the lymphatic vessels along the basilic vein. Intraoperatively, lymphatics can be identified in the superficial subcutaneous tissue with the uptake of patent blue dye. Venules that are of similar vessel diameter, proximity, and suitable direction of lie are selected.

LVAs are performed with 12–0 or 12–0s nylon sutures (CROWNJUN, Japan) in an end-to-end, end-to-side, or multilimbed fashion. Multiple LVAs are performed in each incision if possible. To reduce operative time, we adopt a dual-team approach by performing dLYMPHA alongside the mastectomy or reconstructive team.

## Results

All patients successfully underwent dLYMPHA in Group 2. The average number of LVA anastomoses performed in each limb was 3 (range 1–5 anastomoses). The median number of incisions was 2. The mean duration of surgery was 120 minutes. There were no statistical differences in demographics and patient characteristics between both groups ( Table 1 ). In terms of surgical treatment, the patients in Group 2 (dLYMPHA intervention) had more lymph nodes removed on average than Group 1 (18.2 vs. 14.2, *p* = 0.002), suggesting a more aggressive ALND. In terms of oncological treatment, a higher proportion of patients in Group 2 (dLYMPHA intervention) underwent adjuvant chemotherapy (84.6 vs. 50.6%, *p* = 0.0005), suggesting that the patients in Group 2 may have a higher proportion of aggressive cancers. The rest of the oncological treatment modalities such as adjuvant radiotherapy, neoadjuvant chemotherapy, and neoadjuvant radiotherapy were not significantly different between the two groups. Postoperatively, the incidence of lymphedema was 17.2% (15 patients) and 3.84% (1 patient) in Groups 1 and 2, respectively ( *p* = 0.018). The average follow-up period for all patients was 38.5 months.

The numbers needed to treat (NNT) to prevent one case of lymphedema was calculated using the following formulas:

NNT = 1/absolute risk reduction (ARR)

where, ARR = Control event rate − Experimental event rate

In our study, the number needed to treat to prevent one case of lymphedema was 7 (95% confidence interval of 4–32).

## Discussion

According to the World Health Organization Global Breast Cancer Initiative, breast cancer is the most common cancer afflicting women. Advancements in early detection and treatment have increased life-expectancies with 5-year survival rates exceeding 90% in high-income countries. BCRL is the largest cancer survivorship burden for patients. It is associated with long-term impairments in health-related quality of life in the physical and psychosocial domains up to 10 years postoperatively. 5 Patients commonly experience impairments in upper limb mobility due to arm swelling, tightness, stiffness, and pain. The symptomatic sequelae negatively impact their body image, mental health, and limit engagement in social functions. The incidence of BCRL is 14.1% for patients who undergo ALND, and even higher at 33.4% for those who undergo adjuvant radiation. 2 The prevalence of BCRL will continue to increase in tandem with increasing survival rates. In a chronic incurable disease like BCRL, prevention is better than cure.

The surgical approach to nodal metastasis in breast cancer has undergone numerous evolutions to reduce morbidity and BCRL rates related to ALND. The advent of sentinel lymph node biopsy (SLNB) mitigated unnecessary ALND in clinically node-negative patients and drastically reduced the rates of lymphedema in this group of patients. 6 The American College of Surgeons Oncology Group Z0011 (ACOSOG Z-0011) and EORTC 10981-22023 AMAROS trials demonstrated that patients with one or two metastatic sentinel nodes after SLNB could safely avoid a completion ALND with the use of adjuvant radiotherapy. 7 8 However, in clinically node-positive patients, surgical clearance of the axillary nodes remains the standard of care. Coupled with adjuvant radiation, these often resulted in lymphedema rates of up to 33%. 9

The concept of ILR was introduced by Boccardo in 2009 as the LYMPHA technique. It comprises LVA between the axillary lymphatics and branches of the axillary vein. Long-term outcomes of the LYMPHA technique demonstrated a 4.05% incidence of lymphedema over a 4-year follow-up. 10 There is a growing number of evidence that has suggested the efficacy of this procedure. A meta-analysis by Johnson et al in 2019 showed that the pooled cumulative incidence of lymphedema was significantly lower in patients who underwent ALND with LYMPHA (2.1 vs. 14.1%, *p* = 0.029). 2 Another meta-analysis by Hill et al in 2022 showed similar results favoring lymphedema reduction with LYMPHA (6.7% of patients in the LYMPHA group vs. 34% of patients in the control group developed lymphedema). 11 However, there is little understanding of the oncologic safety of ILR after ALND, especially with the reconstruction done within a cancer field.

The basis for oncologic concern of ILR in the axilla stems from the theory that preserved lymphatics afferent to cancer-containing axillary lymph nodes are anastomosed to the systemic circulation via the LVAs. Remnant microscopic disease may be left behind along these afferent channels due to the proximity to axillary nodes. We are unable to reliably compare the oncologic safety between LYMPHA and dLYMPHA due to the paucity of long-term data, 12 13 but by performing the LVAs distal to the axilla, we can safely avoid any theoretical iatrogenicity in cancer recurrence while providing similar rates of lymphedema incidence (3.84%, *p* = 0.018).

Furthermore, the axilla is a target site for adjuvant radiotherapy and the LVA in LYMPHA is often directly irradiated. Even when it is not deliberately targeted, it receives substantial radiation dosages of up to 4,961 cGy. 14 Impairment in lymphangiogenesis 15 and subsequent fibrosis caused by radiotherapy may affect the patency of any LVA performed. In the dLYMPHA technique, we prefer to perform at least two LVAs at separate sites on the bicipital groove and dorsoradial wrist crease. This puts the LVAs outside the zone of radiation, avoiding the possibility of future stenosis from radiation-related fibrosis. In addition, the large lymphatic vessels of the upper limb run along the cephalic and basilic venous systems. Performing LVAs to the dorsoradial wrist crease and bicipital grove, it allows the decompression of the cephalic and basilic-related lymphatic vessels, respectively. Thus, this allowed for efficient decompression of the upper limb below and above the elbow joint, respectively, and anterolateral and anteromedial lymphangiosomes, respectively. 16

There exists rich and variable lymphatic pathways along the anterior and posterior locations of the upper limb. 17 They all work to drain the upper limb into the axilla. Lymphatic channels are widely available and similar-sized venules with lower venous pressures are typically adjacent and easy to locate. Better vessel match consequently translates to improved patency. In contrast to reports utilizing the LYMPHA technique, we did not encounter any situation where dLYMPHA was not suitable in the upper limb. Recognizing similar shortcomings of the LYMPHA technique and advantages of moving the LVA distally, Orfahli et al proposed the use of a distally based LVA. 18 However, due to scheduling constraints, they were unable to coordinate the oncologic resection and lymphatic reconstruction into one event. We did not face these logistical constraints in our center and were able to perform surgery concurrently with the breast resection or reconstruction, thereby reducing operative time. We routinely deploy two microscopes and two microsurgical teams, if necessary to allow the lymphatic and breast reconstruction to occur simultaneously ( Fig. 4 ). To optimize space management between the lymphatic reconstructive and breast team, we perform the dLYMPHA with an arm board extension from the operating table. Additionally, the dLYMPHA technique is performed with the surgeon positioned perpendicularly to the arm, allowing for a more ergonomic posture, and allowing the participation of a surgical assistant seated opposite. In the classic LYMPHA technique, the surgeon would have to position himself diagonally oblique to the axilla to perform the LVA, obviating the possibility of having a surgical assistant.

In terms of potential risk factors, obesity has been associated with increased risks of lymphedema. 19 However, obesity is itself not a causative factor for lymphedema unless it is at the extreme level where the body mass index (BMI; kg/m ^2^ ) is more than 40. In our cohort, the mean BMIs for both treatment (dLYMPHA) and nontreatment groups are similar at 25.2 and 24.0, respectively, with no statistically significant differences ( *p* = 0.296). This showed that lymphedema can occur in our patients even when they are at a normal or near-normal BMI of 25. In addition, even when performed in normal BMI patients, dLYMPHA can reduce the risk of BCRL. Therefore, our group is of the opinion that high BMI should not be used as an inclusion criterion for dLYMPHA, and neither is normal BMI an exclusion criterion for dLYMPHA.

Another potential risk factor for the development of BCRL is the number of lymph nodes retrieved and the aggressiveness of breast disease. In our study, the number of lymph nodes removed was higher in the dLYMPHA group (18.2 vs. 14.2, *p* = 0.002). Despite this, the rate of lymphedema was still lower in the dLYMPHA group. In addition, there was a higher percentage of patients undergoing adjuvant chemotherapy in the dLYMPHA group (84.6 vs. 50.6%, *p* = 0.0005). This highlights a few points that strongly support the dLYMPHA technique. Firstly, when the resecting surgeon knows that there will be lymphatic intervention, they can confidently perform a more thorough ALND to achieve R0 margins, and not be adversely influenced by the fear of causing BCRL. Secondly, dLYMPHA demonstrates its effectiveness even in the presence of a more extensive axillary lymphatic disruption. Thirdly, despite the presence of a more aggressive disease that requires adjuvant chemotherapy, dLYMPHA could still reduce the risk of BCRL.

In our cohort, the NNT to prevent one case of lymphedema was seven patients. This was similar in the use of low-dose, low-molecular-weight heparin to prevent deep vein thrombosis. 20 This suggests that the dLYMPHA technique has a good risk-benefit/risk ratio in the prevention of BCRL.

Though a better size match can be achieved between lymphatics and venules in the upper limb, the biggest challenge of the dLYMPHA technique is finding suitable venules that are in proximity and the technical difficulty of performing the anastomoses. In our series, there was no prior pathological dilatation of the lymphatic vessels from lymphedema, and due to their distal locations, the diameter of the lymphatic vessels only ranged from 0.15 to 0.30 mm. Unlike the lymphatic channels of secondary lymphedema that are already dilated due to lymphatic obstruction, these lymphatic channels are at normopressure, transparent, and difficult to dilate with forceps. Hence, they can pose significant technical difficulties. In order to ensure good long-term outcomes, any potential risk of anastomotic failure must be prevented. Selection of anastomotic locations, best size match, and mastery of supermicrosurgical techniques will help to improve patency and success rates. 19 Another disadvantage of the dLYMPHA technique was the presence of additional scars distal to the axilla. We limited the size of our incisions to 2 cm and preferentially placed them on the medial bicipital groove and on the distal wrist, where they can be hidden by sleeves and watch straps, respectively ( Fig 3 ). dLYMPHA demonstrated good potential in the primary prevention of BCRL and is an alternative to its predecessor, the LYMPHA technique. Further research is required to establish the long-term results and oncological safety of both the LYMPHA and dLYMPHA techniques.
